# Improving knowledge and confidence in the acute management of eating disorders and resulting complications

**DOI:** 10.1192/bjo.2021.503

**Published:** 2021-06-18

**Authors:** Sarah Fynes-Clinton, Clare Price, Louisa Beckford, Maisha Shahjahan, Brendan McKeown

**Affiliations:** Coventry and Warwickshire Partnership Trust

## Abstract

**Aims:**

This project aimed to improve the knowledge and confidence of doctors at all levels when managing patients with eating disorders while on call.

**Background:**

A recent survey found just 1% of doctors have the opportunity for clinical experience on eating disorders. Anecdotally, a number of junior doctors within our trust had mentioned that they felt unsure when asked to manage patients with eating disorders during their out of hours shifts.

**Method:**

This project aimed to ascertain levels of confidence with managing patients with eating disorders, and to collect suggestions to improve this. This was achieved using a survey sent out to 97 doctors working in a Mental Health Trust.

We then utilised two of the suggestions to improve the identified areas of concern. The first method involved direct lectures. This was followed up with the creation of a poster highlighting the pertinent information which was displayed in key clinical areas. The second avenue was the creation of an information booklet covering key clinical information that is available to all on call doctors.

**Result:**

The response rate for the survey was 37.11%. The survey found that doctors lacked confidence in the management of common conditions that arise in patients admitted with eating disorders. Refeeding syndrome was identified as the greatest area of concern by responding doctors.

To assess the impact of the lectures, MCQs were given out before and after the presentation. The results were compared, and showed a clear improvement in overall knowledge, with results going from an average score of 56.6% to 80%.

**Conclusion:**

By using multiple methods to improve doctors confidence, (lectures, written information and visual posters), this quality improvement project achieved its aims in improving doctors knowledge, and through having easy access to important information, will have long term positive effects on patient care.

